# Efficacy of Nalbuphine with Flurbiprofen on Multimodal Analgesia with Transverse Abdominis Plane Block in Elderly Patients Undergoing Open Gastrointestinal Surgery: A Randomized, Controlled, Double-Blinded Trial

**DOI:** 10.1155/2018/3637013

**Published:** 2018-01-28

**Authors:** Yu Mao, Yuanyuan Cao, Bin Mei, Lijian Chen, Xuesheng Liu, Zhi Zhang, Erwei Gu

**Affiliations:** ^1^Key Laboratory of Brain Function and Disease of Chinese Academy of Science, Department of Biophysics and Neurobiology, University of Science and Technology of China, Hefei, Anhui 230027, China; ^2^Department of Anesthesiology, First Affiliated Hospital of Anhui Medical University, Hefei, Anhui 230031, China

## Abstract

**Objective:**

To assess different doses of nalbuphine with flurbiprofen compared to sufentanil with flurbiprofen in multimodal analgesia efficacy for elderly patients undergoing gastrointestinal surgery with a transverse abdominis plane block (TAPB).

**Methods:**

158 elderly patients scheduling for elective open gastrointestinal surgery under general anesthesia and TAPB were randomly assigned to four groups according to different doses of nalbuphine with flurbiprofen in postoperative intravenous analgesia (PCIA). Postoperative pain intensity, effective pressing numbers of PCIA, and adverse effects were recorded at 6, 12, 24, and 48 hours after surgery.

**Results:**

Postoperative pain intensity, effective pressing numbers, and the incidence of postoperative nausea and vomiting (PONV) were similar among the four groups after surgery, while the severity of PONV was decreased in Group L compared with Group S at 6, 12, and 48 h after surgery. No individual experienced pruritus, respiratory depression, or hypotension.

**Conclusions:**

Low dose of nalbuphine (15 *μ*g·kg^−1^·ml^−1^) combined with flurbiprofen is superior for elderly patients undergoing elective open gastrointestinal surgery with TAPB in terms of the efficient postoperative analgesia and decreased severity of PONV. This trial is registered with NCT02984865.

## 1. Introduction

Abdominal surgeries have greater postoperative mortality and complications that are correlated with increased age [1]. Especially, 75% of postoperative patients experience inadequate pain after gastrointestinal surgery, and uncontrolled postoperative pain prompts respiratory distress, delays wound healing, and a potentially eventual transition from acute to chronic pain problems [2–4]. Thus, it severely challenges the proper use of analgesics for elderly patients undergoing open gastrointestinal surgery to clinicians.

Mu opioid receptor agonists are the most common analgesics for postoperative pain management; however, they have severely adverse effects such as pruritus, respiratory depression, postoperative nausea and vomiting (PONV), urinary retention, constipation, bradycardia, and hypotension [5], which increase postoperative morbidity and mortality in elderly patients mainly due to decreased physiologic reserves, age-related comorbidities, altered pharmacodynamics, and pharmacokinetics [6, 7]. Nalbuphine, a narcotic kappa receptor agonist and partial mu receptor antagonist, provides comparable analgesic efficacy to morphine by modulating visceral pain [8] but with fewer opioid-induced adverse effects [9]. Unfortunately, limited data propose a putative promise of nalbuphine in the treatment of pain for elderly patients undergoing open gastrointestinal surgery.

Importantly, multimodal analgesia is defined as combining analgesics with different mechanisms of action within the peripheral and central nervous system to maximize analgesic efficacy and minimize side effects [10]. Opioids plus nonsteroidal anti-inflammatory drugs (NSAIDs) are associated with greater patient satisfaction and reduce opioid use with fewer opioid-induced adverse effects [11]. Flurbiprofen axetil, a nonselective NSAID incorporated in lipid microspheres, has high affinity for inflammatory tissue and surgical incision sites to control postoperative pain by blocking cyclooxygenase. In addition, preoperative and postoperative administration of flurbiprofen reduced postoperative opioid consumption and decreased systemic proinflammation [12]. However, the postoperative analgesic efficacy of nalbuphine in combination with flurbiprofen is not clear in the elderly patients undergoing open gastrointestinal surgery. Moreover, the transverse abdominis plane block (TAPB) is a relatively novel procedure for applying local anesthetics into the anatomic neurofascial space and provides analgesia to the skin, muscles of the anterior abdominal wall, and parietal peritoneum. Specifically, it offers a significantly prolonged duration of analgesia during the early postoperative stage [13, 14], and reduces opioid consumption after abdominal surgery [15], suggesting the potential use for elderly patients undergoing open gastrointestinal surgery.

In the randomized, controlled, and double-blinded clinical trial, we investigate the postoperative analgesic efficacy and side effects of nalbuphine plus flurbiprofen compared to those of sufentanil plus flurbiprofen. Specifically, the study aimed to test the joint hypothesis that nalbuphine combined with flurbiprofen as postoperative analgesia will provide effective analgesia control and reduce side effects of postoperative opioid consumption compared with sufentanil added with flurbiprofen in elderly patients undergoing open gastrointestinal surgery based with TAPB.

## 2. Materials and Methods

### 2.1. Protocol

The search protocol was approved by the Ethics Committee of the First Affiliated Hospital of Anhui Medical University (PJ-2016-09-05) and registered at ClinicalTrials.gov (NCT02984865; principal investigator: Yu Mao; registration: November 11, 2016). We conducted a randomized, controlled, and double-blinded clinical trial in elderly patients undergoing elective, open gastrointestinal surgery from December 2016 to June 2017 at a single site. This study report was prepared in accordance with the Consolidated Standards of Reporting Trials (CONSORT) guidelines [16, 17].

### 2.2. Study Participants

Patients were included if they were aged 65 years or older, with an ASA physical status of I–III, and able to understand the study and communicate with the study team. Eligible surgical procedures included radical gastrectomy for gastric cancer, radical resection of rectal carcinoma, or colon cancer. Patients were interviewed before the day of surgery, and informed consent was obtained from each patient before entry into the study. Subjects were excluded if they had a serious coexisting disease (e.g., respiratory insufficiency, class II–IV organic heart disease, history of recent brain injury, and hepatic or renal impairment) or a history of multiple adverse drug reactions or an allergy to anesthetic drugs or prior treatment with the study drug.

### 2.3. Randomization and Blinding

The randomized numbers were generated in varying block sizes on a 1 : 1 : 1 : 1 ratio without restrictions to any of the four groups using computer and sealed in opaque envelopes by a research coordinator without involvement of further study. The anesthesia assistant got one envelope and prepared all the study solutions after consented participant was transferred to the operation room. The anesthesia assistant was not involved in the further study; patients and anesthesiologists performing anesthesia were blinded to the allocation. All study participants receiving postoperative intravenous analgesia (PCIA) are as follows: Group S (sufentanil): 25 (ng·kg^−1^·ml^−1^) sufentanil plus 1 mg·ml^−1^ flurbiprofen; Group L (low-dose nalbuphine): 15 (*μ*g·kg^−1^·ml^−1^)nalbuphine plus 1 mg·ml^−1^ flurbiprofen; Group M (medium-dose nalbuphine): 20 (*μ*g·kg^−1^·ml^−1^) nalbuphine plus 1 mg·ml^−1^ flurbiprofen; and Group H (high-dose nalbuphine): 25 (*μ*g·kg^−1^·ml^−1^) nalbuphine plus 1 mg·ml^−1^ flurbiprofen. The study solution of PCIA was mixed in 150 ml, and PCIA settings included a 2 ml bolus with a lockout interval of 15 min and a background infusion of 2 ml·h^−1^ without a loading dose.

### 2.4. Preoperative Procedures

Patients were transferred to the operating room without premedication and standard monitoring including arterial blood pressure, electrocardiogram (ECG), pulse oximetry, and bispectral index (BIS) (Vista; Aspect Medical Systems Inc., Norwood, Norfolk County, Massachusettes, USA) was applied in all individuals. Oxygen was given via mask and 5 *μ*g sufentanil was given prior to implementing TAPB.

### 2.5. TAPB Technique

The preoperative TAPB guided by ultrasound was performed by an experienced regional anesthesiologist under sterile conditions. 2% chlorhexidine solution was used to clean the skin and a 100 mm, 22-G needle (Stimuplex A, B-Braun Medical, Bethlehem, Pennsylvania, Germany) was inserted using an in-plane technique guided by a linear, high frequency ranging from 15 to 6 MHz ultrasound probe (M-Turbo®, SonoSite Inc., Brothell, WA, USA) covered with a sterile sheath (3M Tegaderm, St.Paul, Minnesota, USA).

For lower abdominal surgery, the transducer was placed transversely in the region of the anterior axillary line between the costal margin and the iliac crest. Once the three muscular layers of the abdominal wall (the external oblique, the internal oblique, and the transverse abdominis muscle) were identified, the neurofascial plane between the internal oblique and the transverse abdominis muscle was recognized. For upper abdominal surgery, the probe was placed just oblique to the sagittal plane and inferior to the costal margin to identify the lateral border of rectus muscle and the medial border of transverse abdominis [18]. Once the tip of the needle was placed into the neurofascial plane between the transverse abdominis and the internal oblique muscle or between the posterior rectus sheath and transverse abdominis, a small amount of fluid (1–2 ml saline) was injected to hydrodissect the appropriate plane. Then, local anesthetic (15 ml of 0.5% ropivacaine and 5 mg dexamethasone) was deposited into the fascial layer to separate the internal oblique and transverse abdominis muscles. The injection appeared as a dark oval under ultrasound. Bilateral TAPB was applied for midline incision. TAPB was considered successful when the surgical site was hypoalgesic or analgesic as measured with acupuncture pain detection 10 min after TAPB. Block failure was noted for patients when block success was not achieved after 30 min, and the data were deleted in the analysis.

### 2.6. General Anesthesia

A standardized general anesthesia in all participants was administrated by an anesthesiologist blinded to randomized allocation. Propofol was administrated using a target-controlled infusion (TCI) pump (Graseby 3500 Anesthesia Pump; Graseby Medical Ltd., United Kingdom) during the induction of anesthesia. After the initial target concentration of 1.0 *μ*g/ml was reached, the concentration was gradually increased by 0.3 *μ*g·ml^−1^ until the BIS value was <60. Sufentanil (0.3–0.5 *μ*g·kg^−1^ and cisatracurium (0.2 mg·kg^−1^) was injected and followed by tracheal intubation. End-tidal carbon dioxide was continuously monitored after intubation. Tidal volume and ventilation rate were adjusted with 100 percent oxygen to maintain the end-tidal CO_2_ partial pressure of arterial blood at 35–45 mmHg. The TCI of propofol was continuously infused to maintain BIS values from 45 to 60, and 0.1 mg·kg^−^1 cisatracurium were intermittently injected according to need. 0.1–0.2 *μ*g·kg^−1^ sufentanil was titrated for analgesia, as needed, if heart rate (HR) and/or mean arterial pressure (MAP) increased by 20% above baseline during surgery. Flurbiprofen (50 mg, iv) was administered before skin incision, and 0.1 *μ*g·kg^−1^ sufentanil plus 50 mg flurbiprofen in group S or 0.1 mg/kg nalbuphine plus 50 mg flurbiprofen in L, M, and H groups were given intravenously followed by a PCIA pump just before the peritoneum was closed. Neostigmine (20 *μ*g/kg) and atropine (5–10 *μ*g/kg) were administrated intravenously to reverse residual muscle relaxation when spontaneous respiratory recovered at the end of surgery. Bradycardia or hypotension encountered during surgery or recovery was treated on the basis of the following algorithm: bradycardia (HR decreases by 20% from baseline), 0.2–0.3 mg IV atropine; hypotension (MAP decreases by 20% from baseline), 40 *μ*g IV phenylephrine; and bradycardia and hypotension, 3–6 mg IV ephedrine. All participants received the standard surgical procedures determined by the surgeons, and surgical management in the study was not altered in any way.

### 2.7. Postoperative Management

Patients were moved to the postanesthesia care unit (PACU) after surgery and extubated until they met extubation criteria. A bolus of intravenous analgesia of PCIA pump was allowed to give by a blinded PACU nursing staff when a 10 com visual analogue scale (VAS) value for pain exceeded 4. Patients were then transferred to wards when Steward Recovery Value exceeded 4. Patients were followed up by a nurse blinded to group allocations at 6, 12, 24, and 48 h as well as 7 days after surgery.

### 2.8. Outcome Measures

The primary outcome was the incidence of PONV at 6, 12, 24, and 48 h postoperatively. The degree of PONV was stratified as follows: 0, no nausea and vomiting; 1, mild; 2, moderate; 3, severe. Ondansetron (4 mg, iv) was given at the patient's request to treat PONV.

Secondary outcomes included (1) a 10 cm VAS for pain (0, no pain; 10, worst imaginable pain); (2) effective pressing times of PCIA; (3) Ramsay sedation score (1, anxious, agitated, or restless; 2, cooperative, oriented, and tranquil; 3, responsive to command; 4, briskly responsive; 5, a sluggish response; 6, no response); (4) heart rate, noninvasive arterial pressure, respiratory rate, and oxygen saturation; (5) side effects (pruritus, respiratory depression, and hypotension) at 6, 12, 24, and 48 h postoperatively; and (6) short-time recovery (the first postoperative day for leaving the bed and intestinal movement, postoperative hospital duration, and hospitalization cost).

Skin pruritus was recorded by incidence and diphenhydramine (25 mg, po) was given on demand. Respiratory depression was described as respiration <8 bpm or oxygen saturation <90%. Supplemental oxygen therapy was administrated to increase the fraction of inspired oxygen. PCIA would be stopped immediately if oxygen therapy was invalid. Naloxone (0.1 mg/kg) was available if needed to rescue respiration inhibition due to oversedation. IV fluids and closely monitoring were applied to treat hypotension defined as MAP decreased by 20% from baseline. PCIA would be stopped if blood pressure interventions were not successful.

### 2.9. Statistical Analysis

We aimed to test the joint hypothesis that nalbuphine with flurbiprofen, regardless of different dosages, provides effective postoperative analgesia and reduces side effects of postoperative analgesic consumption compared with sufentanil plus flurbiprofen in elderly patients experienced open gastrointestinal surgery with TAPB and to successively examine whether this multimodal strategy is an effective postoperative pain management with fewer side effects. Consequently, we started by comparing the PONV postoperatively, and sample size was calculated with previous data that were collected from 20 patients undergoing open gastrointestinal surgeries with general anesthesia-based TAPB using sufentanil and flurbiprofen as postoperative analgesia. The incidence of PONV 6 h after surgery was 0.35 in the previous study. Keeping a 5% type 1 error rate between groups and a study power of 80%, 31 subjects per group at a minimum were calculated to identify a difference of 0.25 at PONV 6 h after surgery.

Data were analyzed using the Statistical Package for the Social Sciences program (SPSS 19.0). Quantitative data were presented as mean (SD) or mean (95% CI), and categorical data were showed as numbers or percentages. Quantitative data were analyzed using one-way ANOVA, and an LSD procedure was applied for post hoc comparisons. PONV severity and Ramsay sedation scale were assessed using a Kruskal–Wallis test. A Mann–Whitney *U* test was used for intergroup comparisons when a significant difference was found among groups. The incidence of PONV was analyzed using a chi-square test or a Fisher's exact test (*p* < 0.05 was considered statistically significant). Bonferroni corrections were applied to correct for multiple comparisons testing (*p* < 0.01 was considered statistically significant).

## 3. Results

### 3.1. Participants Flow

**Figure 1:**
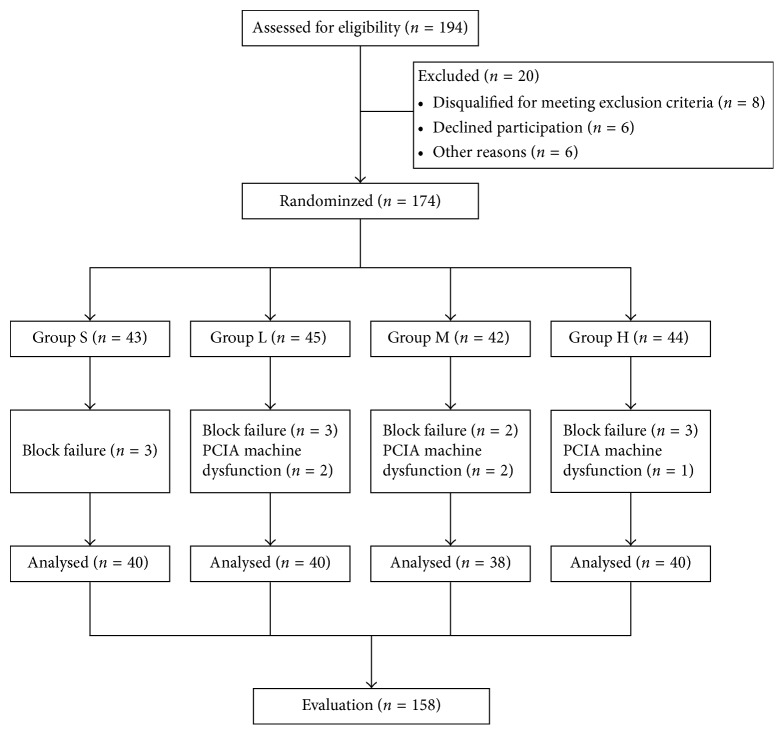
Flow of patients in the study.

Figure 1 depicts the CONSORT flow of participants through the trial. 194 individuals were assessed for the eligibility of the study, of these 14 either disqualified for meeting exclusion criteria or individuals who met the inclusion criteria declined to participate in the study, 6 declined for other reasons. A total of 174 individuals were randomized: 43 were assigned to Group S, 45 to Group L, 42 to Group M, and 44 to Group H. 3 individuals in Group S, 5 in Group L, 4 in Group M, and 4 in Group H were excluded in the trial due to block failure or PCIA machine dysfunction, and 158 individuals finally completed the study.

Basic subject characteristics appear in Table 1. There were no significant differences among groups in terms of gender, ASA, age, weight, height, BMI, and MAP. Anesthesia duration, operation time, consumption of sufentanil, crystalloid fluid infusion, and colloid fluid infusion during operation were not statistically significant different among groups, while awake time and extubation time during recovery were not significantly different among groups (Table 2).

### 3.2. PCIA Requirements and Pain Intensity

Effective PCIA machine pressure times and pain VAS data were not significantly different among the four groups during the observation period (Table 3). No patient experienced insufficient analgesia or treatment with adjunctive analgesics in this trial.

### 3.3. PCIA-Related Adverse Events

The incidence of postoperative nausea and vomiting was not significantly different among the four groups. PONV severity was significantly different among the four groups at 6, 12, and 48 h after surgery according to a Kruskal–Wallis test (*p*=0.002, 0.001, and0.029, resp.; Table 4). PONV severity was statically different in group S compared with group L at 6, 12, and 48 h after surgery (*p*1=0.002, 0.002, and0.006, resp.). The Ramsay scores of sedation were similar among four groups (Table 5). Only one patient in the group H reported that Ramsay score was 5 when assessed at 24 and 48 h, which resolved completely after the PCIA infusion finished. No patient experienced pruritus, respiratory depression, or hypotension in this study.

### 3.4. Postoperative Short-Time Recovery

The first day for bed-leaving activity and intestinal movement, postoperative hospital duration, and hospitalization expense were not significantly different among any group (Table 6).

## 4. Discussion

Appropriate perioperative analgesia is a fundamental component of enhanced recovery after surgery [19, 20]. Although epidural anesthesia is the standard care for postoperative pain, it has contraindications and limitations, such as spinal hematoma, epidural abscess, and hypotension and technical complications, especially for elderly patients who frequently take antiplatelets [21–23]. A meta-analysis indicated that compared with alternative analgesic techniques, epidural analgesia did not provide additional clinical benefits to patients during laparoscopic colorectal surgery [24]. TAPB is a novel and effective analgesia for controlling postoperative pain, and it can provide somatic anesthesia for abdominal surgeries [25–29]. It is important to note that the shortcoming of a single injection of local anesthetic is the limited time of regional neural blockade [30]. Dexamethasone, yet not approved by FDA as an adjunct to local anesthetics, was still demonstrated to prolong the duration of analgesia after peripheral nerve blockade [31]. Because the potential for toxicity is increased with higher doses of local anesthetic [32, 33], 30 ml of 0.5% ropivacaine and 10 mg of dexamethasone as supported by the literature [29, 33] was the maximum volume used in this study. No toxicity or adverse events were observed in elderly patients, suggesting that 30 ml of 0.5% ropivacaine and 10 mg dexamethasone can be safe.

Even though successful TAPB can provide an effective anesthetic for abdominal somatalgia after surgery, it cannot provide complete postoperative analgesia for intraperitoneal surgeries, as it does not address pain in the abdominal viscera [29]. NSAIDs modulate pain pathways by reducing local inflammation and preventing peripheral and central sensitization [34]. When NSAIDs are administrated intravenously, they act as an adjunct regional blockade by suppressing prostaglandin E2 and cytokines in addition to inhibiting neural responses to noxious injury [35]. Flurbiprofen injection encapsulated in lipid microspheres is a NSAID that decreases opioid consumption and offers better postoperative analgesia for patients after spinal fusion surgery [11, 36]. Because NSAIDs pose a risk of anastomotic leakage [37–39], flurbiprofen should be given in low doses and combined with opioids to provide effective analgesia and decrease the risk of anastomotic leakage [40]. Mu opioid receptor agonists act at the central nerve system and the gastrointestinal tract [8] and as such may be associated with adverse effects. Peripheral kappa opioid receptors, present in the visceral afferents of the gastrointestinal tract [8], are critical modulators of visceral pain. Nalbuphine agonizes the kappa receptor to mediate visceral pain and potentially antagonizes the mu receptor to attenuate adverse effects of mu agonists. Hence, nalbuphine is of interest as a compound that offers pain relief and few side effects.

Potency at mu 1 and 2 and kappa receptors is sufentanil > nalbuphine, sufentanil > nalbuphine, and nalbuphine >> sufentanil [41, 42]. Agonism at the mu 1 and 2 receptors produces analgesia and respiratory depression, respectively, and agonism at the kappa receptor produces analgesia and sedation. Therefore, nalbuphine is superior to morphine and offers comparable analgesia and few adverse effects [9]. Because the analgesic potency of morphine is equivalent to that of nalbuphine, the ratio of an equipotent dose of sufentanil and nalbuphine is 1000 : 1 [9, 43, 44]. In this study, sufentanil was delivered (iv, PCIA machine) at 25 (ng·kg^−1^·ml^−1^) to group S, and nalbuphine was 15 (*μ*g·kg^−1^·ml^−1^), 20 (*μ*g·kg^−1^·ml^−1^), and 25 (*μ*g·kg^−1^·ml^−1^) to L, M, and H groups, respectively. Our data reveal that low dose of nalbuphine (15 *μ*g·kg^−1^·ml^−1^) provides equivalent postoperative analgesia compared to high dose of sufentanil (25 n·g·kg^−1^·ml^−1^), likely because kappa receptor agonism blocking visceral pain is better than mu receptor agonism [8, 45]. Increasing the nalbuphine does not increase the analgesic efficacy, suggesting a ceiling effect for analgesia as well as respiratory depression [43]. PONV is as high as 20–30% of the general surgical population, and postoperative opioids consumption is a risk factor for PONV [46]. Even though the mechanism of PONV occurring after mu receptor agonist use is unclear [47, 48], the severity of PONV is significantly decreased in group L compared with group S at 6, 12, and 48 h after surgery. Therefore, low dose of nalbuphine is better than high dose of sufentanil with equivalent postoperative analgesia and decreased severity of PONV.

It should be noted that 10–50% of patients treated with intravenous opioids acquired opioid-induced pruritus (OIP) [49] and 1.1% of patients receiving postoperative opioid analgesia experienced respiratory depression [50], but this did not occur with any elderly patient in this study. Since the incidence of pruritus and ventilation abnormalities are dose-dependent [51], Group S outcomes may be due to low-dose sufentanil with TAPB. Because nalbuphine antagonizes the mu receptor, it is recommended as a first-line treatment for OIP [47], and respiratory depression was not noted at any dose of nalbuphine during the observation.

In summary, using multimodal analgesia with TAPB for elderly patients undergoing gastrointestinal surgery, we demonstrated that the optimal combination for PCIA is low dose of nalbuphine (15 *μ*g·kg^−1^·ml^−1^) and flurbiprofen (1 mg·ml^−1^) to minimize severity of PONV compared to PCIA with sufentanil and flurbiprofen after surgery. This study may provide a novel and optical option of multimodal analgesia for enhanced recovery in elderly patients undergoing gastrointestinal surgery with fewer side effects.

## Figures and Tables

**Table 1:** Basic characteristics of patients.

	Group S	Group L	Group M	Group H	*p*
Gender (male/female)	30/10	29/11	27/11	28/12	0.279
ASA (I/II/III)	3/27/10	5/34/1	4/23/11	2/29/9	0.070
Age, mean (SD) (yr)	71.40 (4.65)	71.15 (5.60)	70.15 (4.46)	71.63 (5.79)	0.592
Weight, mean (SD) (kg)	59.60 (9.39)	57.53 (8.39)	57.85 (10.19)	60.93 (11.21)	0.377
Height, mean (SD) (m)	1.65 (0.68)	1.65 (0.71)	1.63 (0.70)	1.63 (0.85)	0.627
BMI, mean (SD)	21.81 (2.87)	21.25 (2.71)	21.68 (3.77)	22.83 (3.66)	0.179
MAP, mean (SD) (mmHg)	105.2 (12.4)	102.1 (13.0)	99.2 (15.5)	103.6 (14.2)	0.257

BMI = body mass index.

**Table 2:** Characteristics of surgery and anesthesia during operation and recovery.

	Group S	Group L	Group M	Group H	*p*
Anesthesia duration (min)	155.80 (48.86)	162.83 (49.79)	169.40 (57.75)	172.78 (54.66)	0.493
Operation time (min)	134.18 (45.73)	139.60 (46.37)	144.35 (52.60)	153.08 (56.58)	0.392
Sulfentanyl (*μ*g)	48.50 (12.51)	46.58 (9.75)	49.69 (10.16)	50.56 (12.50)	0.427
Crystalloid fluid (ml)	1135.00 (395.84)	1166.67 (555.04)	1150.00 (451.78)	1198.75 (415.48)	0.956
Colloid fluid (ml)	510.00 (271.79)	578.95 (184.77)	512.50 (232.80)	487.50 (306.50)	0.435
Awake time (min)	30.51 (23.93)	30.35 (20.00)	35.65 (27.93)	30.40 (20.71)	0.682
Extubation time (min)	28.28 (18.60)	28.88 (17.22)	31.98 (19.33)	28.48 (20.50)	0.801

**Table 3:** The VAS pain score and effective pressing numbers of PCIA machine during the observation period.

	Observational period	Group S	Group L	Group M	Group H	*p*
VAS pain score	0–6 h	1.05 (1.449)	0.95 (1.011)	0.98 (1.223)	0.98 (1.365)	0.988
	6–12 h	1.40 (1.598)	1.00 (0.987)	1.05 (1.260)	1.23 (1.423)	0.527
	12–24 h	1.75 (1.721)	1.28 (0.176)	1.18 (1.130)	1.08 (1.403)	0.084
	24–48 h	1.68 (1.685)	1.30 (1.305)	1.18 (1.238)	1.20 (1.418)	0.363
Effective pressing numbers	0–6 h	0.55 (1.108)	0.60 (1.008)	0.88 (1.453)	0.83 (1.708)	0.620
	6–12 h	0.75 (1.463)	0.58 (1.357)	1.00 (1.617)	0.65 (1.099)	0.558
	12–24 h	1.95 (5.228)	1.18 (2.531)	1.30 (2.151)	0.73 (1.633)	0.399
	24–48 h	1.28 (1.679)	1.30 (2.775)	1.03 (1.459)	0.55 (0.783)	0.225

VAS = visual analogue scale.

**Table 4:** The incidence and severity of postoperative nausea and vomiting during the observation period.

	Group S	Group L	Group M	Group H	*p*	*p*1	*p*2	*p*3
Incidence (n/N)	—	—	—	—	—	—	—	—
0–6 h	9 (40)	0 (40)	2 (38)	2 (40)	0.238	—	—	—
6–12 h	9 (40)	0 (40)	2 (38)	1 (40)	0.213	—	—	—
12–24 h	7 (40)	1 (40)	3 (38)	3 (40)	0.238	—	—	—
24–48 h	7 (40)	0 (40)	2 (38)	3 (40)	0.213	—	—	—
Severity (0/1/2/3)	—	—	—	—	—	—	—	—
0–6 h	31/7/1/1	40/0/0/0	36/1/1/0	38/1/0/1	0.002^∗^	0.002^∗^	0.033	0.027
6–12 h	31/7/1/1	40/0/0/0	36/1/1/0	39/1/0/0	0.001^∗^	0.002^∗^	0.033	0.007^∗^
12–24 h	33/6/1/0	39/1/0/0	35/2/1/0	37/3/0/0	0.121			
24–48 h	33/6/1/0	40/0/0/0	36/1/1/0	37/3/0/0	0.029^∗^	0.006^∗^	0.103	0.171

*p*1, Group L versus Group S; *p*2, Group M versus Group S; *p*3, Group H versus Group S; ^∗^statistically significant (*p* < 0.01).

**Table 5:** The records of Ramsay during the observation period.

	Group S	Group L	Group M	Group H	*p*
0–6 h (1/2/3/4/5)	1/34/5/0/0	1/29/10/0/0	0/29/8/1/0	3/25/11/1/0	0.467
6–12 h (1/2/3/4/5)	1/34/5/0/0	1/32/7/0/0	0/32/5/1/0	3/26/11/0/0	0.739
12–24 h (1/2/3/4/5)	1/37/2/0/0	0/36/4/0/0	0/34/4/0/0	2/28/9/0/1	0.170
24–48 h (0/1/2/3/4/5)	0/1/38/1/0/0	1/0/35/4/0/0	0/1/33/4/0/0	0/2/29/8/0/1	0.185

**Table 6:** Postoperative short-time recovery.

	Group S	Group L	Group M	Group H	*p*
Leaving bed activity, POD	3.63 (1.675)	3.68 (1.366)	3.70 (1.588)	3.73 (1.694)	0.988
Intestinal movement, POD	3.83 (1.338)	3.68 (1.185)	3.85 (1.145)	3.63 (1.079)	0.527
Postoperative stay in hospital, POD	11.18 (3.071)	10.85 (3.289)	10.76 (3.467)	11.91 (4.957)	0.084
Hospitalization expenses, ¥	45,030 (10,949)	43,896 (11,572)	47,216 (16,141)	44,291 (9,532)	0.363

POD = postoperative day.
